# The effect of posterior lumbar dynamic fixation and intervertebral fusion on paraspinal muscles

**DOI:** 10.1186/s12891-021-04943-w

**Published:** 2021-12-20

**Authors:** Geng-Xiong Lin, Yan-Ming Ma, Yong-Chun Xiao, Dian Xiang, Jian-Xian Luo, Guo-Wei Zhang, Zhi-Sheng Ji, Hong-Sheng Lin

**Affiliations:** 1 Department of Orthopedics, The First Affiliated Hospital of Jinan University, Guangzhou, 510630 China; 2 Department of Spine and Joint, Xiangxi National Hospital of Traditional Chinese Medicine, Jishou, 416000 China

**Keywords:** K-rod, Dynamic internal fixation, PLIF, Lumbar degenerative disease, Paraspinal muscles atrophy, Fatty infiltration

## Abstract

**Background:**

The aim of this study was to analyze the effect of unilateral K-rod dynamic internal fixation on paraspinal muscles for lumbar degenerative diseases.

**Methods:**

This study retrospectively collected 52 patients who underwent lumbar surgery with the K-rod group or PLIF. The operation time, intraoperative blood loss, postoperative drainage volume, postoperative exercise time were compared in the two groups. The visual analog scale (VAS) score and the oswestry dysfunction index (ODI) were employed to evaluate the clinical outcomes. The functional cross-sectional area (FCSA) of the paraspinal muscles and paraspinal muscles fat infiltration were measured to assess on the paraspinal muscles.

**Results:**

As compared with the PLIF group, the operation time, the postoperative time in the field, and the average postoperative hospital stay in the K-rod internal fixation group were significantly shortened. At the last follow-up, both the groups showed significant improvement in the VAS score and ODI. The FCSA atrophy of the upper and lower adjacent segments (UAS and LAS) of the K-rod internal group was significantly less than that of the PLIF group. The extent of increase in the fatty infiltration of the paraspinal muscles in the K-rod group was significantly lesser than that in the PLIF group. The postoperative low back pain of the two groups of patients was significantly positively correlated with the FCSA atrophy.

**Conclusions:**

As compared to PLIF, the posterior lumbar unilateral K-rod dynamic internal fixation showed significantly lesser paraspinal muscle atrophy and fatty infiltration, which were significantly positively correlated with postoperative low back pain.

## Introduction

Posterior lumbar interbody fusion (PLIF) is a proven method to relieve the symptoms of lumbar degenerative diseases and is widely applied in the treatment of lumbar spinal diseases [1, 2]. The technique of PLIF provides sufficient vision to allow surgeons to completely decompress the compressed nerve roots and dural sac directly, which effectively relieves the symptoms of low back pain and leg pain. In addition, PLIF offers a wider area of intervertebral interbody graft bone contact surface with improved load-sharing and adequate access for the complete decompression of the neurons [3, 4]. However, a stress-shielding effect occurs due to the loss of the movement function of the surgical segment resultant from the rigid fixation of the posterior spine and postoperative fusion of the vertebral bodies, leading to accelerated aging of the adjacent segments of the intervertebral disc and adjacent level spinal deterioration (ASD) [5–7]. Furthermore, paraspinal muscle ischemia and denervation often occur due to the involvement of long incision, extensive stripping, and long intraoperative traction of the muscles that leads to postoperative paraspinal muscle atrophy and fatty infiltration [8–11].

Located on both the sides of the spinous process, the paraspinal muscle is one of the core muscles behind the spine; its structural integrity plays the key role in preserving the spine structural stability and the physiological flexor of the thoracolumbar spine [12–14]. Several studies have documented that PLIF inevitably directly stretches the muscles, affects the blood supply and innervation of the muscles, and decreases the vertebral body mobility, which leads to paraspinal muscle atrophy, fatty infiltration, and intractable low back pain [9, 15–18]. To address these concerns, the researchers are constantly exploring new surgical techniques with the aim of reducing the incidence of iatrogenic injuries to the paraspinal muscles, accelerating the postoperative recovery, and reducing the postoperative low back pain [19, 20]. Dynamic stabilization system has been found to decrease adjacent segment degeneration, reduce the loading on the intervertebral disc and improve spinal stability [21, 22]. The K-rod internal fixation system is a relatively novel, dynamic spine stability device with a positive clinical outcome recorded in both short and medium terms, which was consisted of titanium alloy pedicle screws, polyetheretherketone (PEEK) rod and titanium alloy tubes [23]. This intervention can effectively reconstruct the stability of the spine and retain the spinal movement function [23, 24]. In addition, the internal fixation system can provide single-segment or multi-segment non-fusion solutions as well as can be combined with rigid internal fixation to achieve a diversified combination of fusion and non-fusion effects. Theoretically, the posterior lumbar unilateral K-rod dynamic internal fixation not only effectively treats lumbar degenerative diseases but also reduces the injury to the structure of the spine and the paravertebral soft tissues, thereby avoiding damage to the spinal motor unit and reducing the postoperative paraspinal muscular atrophy and fatty infiltration as well as the occurrence of complications such as postoperative residual low back pain.

Thus, the aim of the present study was to compare and analyze the surgical outcomes and paraspinal muscular atrophy with fatty infiltration between posterior unilateral K-rod internal fixation and PLIF in the treatment of lumbar degenerative diseases at our hospital. The primary hypothesis of the present study is that the former has better surgical effects and causes relatively lesser extent of paraspinal muscular atrophy and fatty infiltration compared to the latter.

## Materials and methods

### Study design and patients

This clinical research was a retrospective analysis of 52 patients who had undergone lumbar spinal surgery from May 2015 to December 2018 at our hospital. The study subjects were categorized into 2 groups: K-rod (*n* = 27; 21 men, 6 women) and PLIF (*n* = 25; 13 men, 12 women). The inclusion criteria of the study subjects were as follows: (1) patients who suffered from lumbar disc herniation (LDH), lumbar spinal stenosis (LSS), or lumbar spondylolisthesis and showed typical symptoms and signs consistent with the clear imaging manifestations; (2) patients who had no history of ineffective surgical treatment in the past ≥6 months as well as had no surgical contraindications; (3) patients with no history of spine-related diseases and surgery; and (4) patients who returned to the hospital regularly for professional examination after the operation and were followed up for > 10 months.

### Surgical procedure

All operations were conducted by the same experienced surgeon and in the prone position under general anesthesia. For example, in the L4/5 disc herniation surgery, the surgeon selected a suitable position for performing incision mark, routinely sterilize drapes, sticking the sterile film, making longitudinal incisions of appropriate lengths, and incising the skin, subcutaneous tissues, and back fascia in sequence.

K- rod internal fixation group: The paraspinal muscles was stripped along the side of the spinous process to the deep portion for exposing the L4-affected side vertebral plate to the facet joint. Using the C-arm machine, the surgeon could complete the preparation of the L4- and L5-affected side pedicle screw channels, selected the appropriate size channel system, and established the working channel. The ligamentum flavum was excised directly, the fenestrated lamina was drilled, and the spinal canal was exposed. An assistant pulled the dural sac and nerve root to the healthy side using the nerve root retractor on the opposite side. The protruding nucleus pulposus and intervertebral disc tissues were cleared under the vision and a probe was applied to ensure that the nerve roots and dura mater were not compressed. After achieving sufficient hemostasis, pedicle screws and peek rods were implanted and fixed. A drainage tube was then placed on the affected side, and the incision was closed layer by layer. No treatment was required on the opposite side.

PLIF group: The paraspinous muscles were stripped from the spinous processes using a subperiosteal technique along the laminar on both the sides and extended laterally to the articular processes. With the help of the C-arm machine, pedicle screws were routinely implanted. Then, cutting off the L4/5 and L5/S1 interspinous ligament, the L4 spinous process was bitten and complete laminectomy was performed to expose the spinal canal. The intervertebral disc tissue with nucleus pulposus and the final plate were accordingly removed. An appropriate sized cage filled with the harvested local bone and demineralized bone matrix was then inserted to the intervertebral space and a rigid connecting rod was applied to connect the pedicle screws, followed by compression and locking. The remaining procedure was the same as that for K-rod dynamic internal fixation.

### Postoperative treatment and rehabilitation

Symptomatic and supportive treatments such as anti-infection, swelling, pain relief, and nerve nutrition were routinely performed after the surgery. The dressing was changed every other day, and the sutures were removed 12–14 days after the surgery. On the first day after the surgery, the patients were encouraged to raise their legs and turn over in the bed as an exercise. On the second day after the surgery, the catheter was removed when the drainage volume reached 30–50 mL or when the drainage fluid became clear. Patients were recommended to continue wearing the waist protection for 2–3 months after their discharge and to avoid heavy exercise in the first month of the surgery. Moreover, appropriate functional exercises were prescribed in combination with their self-recovery for 2–3 months after the surgery.

### Postoperative follow-up and outcome measures

Lumbar spine X-rays were reviewed at our hospital 3 and 6 months after the surgery, while lumbar MRI were reviewed 1 year after the surgery. The surgical time, intraoperative blood loss, postoperative drainage volume, postoperative turning to the activity time on the ground, and postoperative hospital-stay duration were recorded for the two groups. The signs and symptoms were evaluated for low back pain based on the VAS score and ODI. At the final follow-up, MRI of the lumbar spine was obtained and the FCSA of the paraspinal muscles as well as the area of fatty infiltration were recorded. The MRI system was a 1.5 Tesla Imaging System T (Signa HDxt 1.5 T GE, USA). Cross-sectional views of lumbar were obtained using a fast spin-echo sequence system for T2WI. The slice width was 4 mm and the inter-slice gap was 1 mm. The acquisition matrix was 320/224. The sequence parameters were a repetition time of 3000 ms and an echo time of 102 ms for T2WI.

**Fig. 1:**
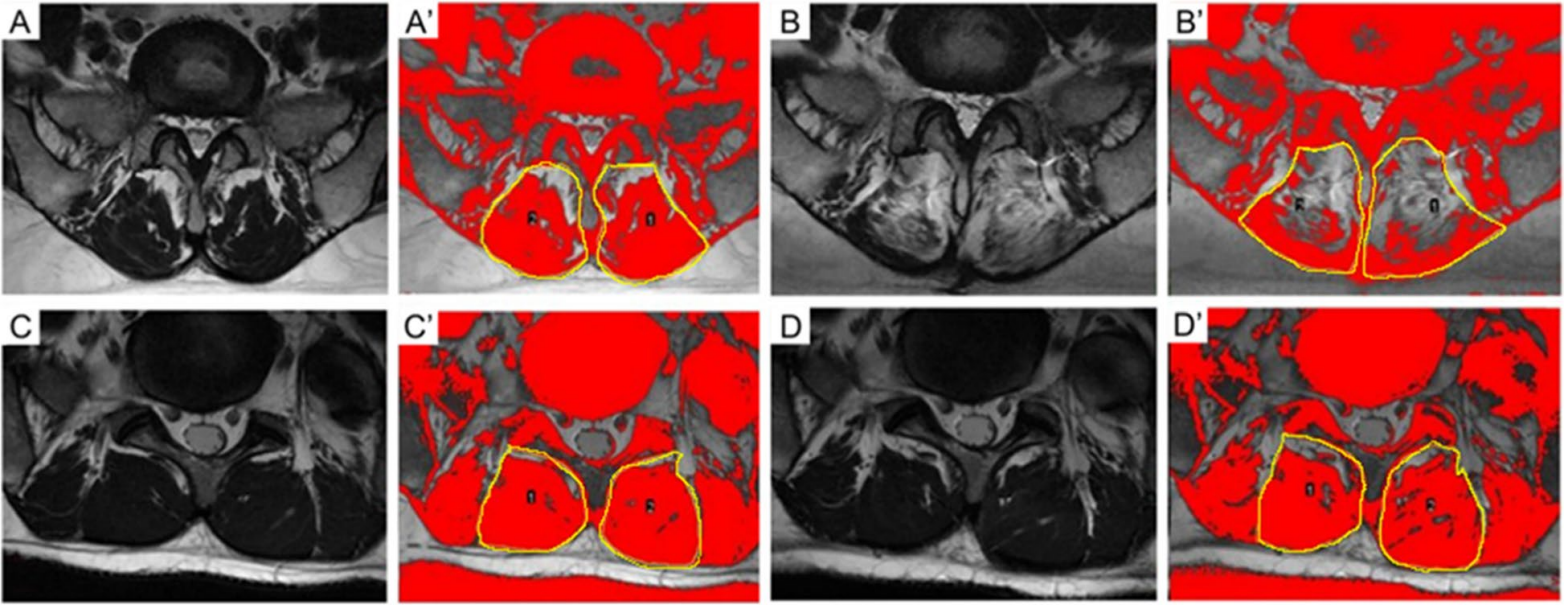
A. MRI T2 Axis map: The preoperative area of paraspinal muscles and fat of LAS in the PLIF group. B. MRI T2 Axis map: The postoperative area of paraspinal muscles and fat of LAS in the PLIF group. C. MRI T2 Axis map: The preoperative area of paraspinal muscles and fat of LAS in the K-rod dynamic internal fixation group. D. MRI T2 Axis map: The postoperative area of paraspinal muscles and fat of LAS in the K-rod dynamic internal fixation group. A’-D′: The periphery of the paraspinal muscles on the image was outlined using yellow line

The measurement of paraspinal muscle FCSA and fatty infiltration acreage: the MRI T2 axial map of the same segment and the same level before and after the operation were selected. Image J software was applied to outline the periphery of the paraspinal muscles on the image with 4X magnification. Then, the boundary of the muscle and adipose tissues was precisely identified and the pure muscle cross-sectional acreage and adipose cross-sectional acreage were calculated (Fig. 1). Two spine surgeon doctors completed the assessment.

### Statistical analysis

All statistical data were analyzed using the SPSS22.0 statistical software (SPSS 109 Inc., Chicago, IL). Quantitative data were indicated as the mean value ± standard deviation (^−^x ± s). The Shapiro-Wilk tests were performed to test the hypothesis of normal distribution. If the date was normally distributed, independent *t-*tests were performed on age, operation time, intraoperative blood loss, postoperative drainage, postoperative exercise time turning to the ground, postoperative hospital stay, VAS score, ODI, and paraspinal muscle FCSA of the two groups. The postoperative and preoperative VAS score, ODI, the paraspinal muscle FCSA, the area of paraspinal muscle fatty infiltration and the paraspinal muscle FCSA of UAS and LAS were tested by two-way (2 × 2) repeated measures analysis of variance; the gender of the two groups was tested by χ^2^ test; the grading change in the paraspinal muscle fatty infiltration of the two group was tested by Fisher’s exact test. *P* < 0.05 was considered to be statistically significant. Bivariate correlation was used to analyze relationships the VAS score of postoperative low back pain and four potential risk factors including the area of fatty infiltration and the area of paraspinal muscle atrophy of UAS and those of LAS. Then, risk factors with significant level were further identified using multivariate linear regression. *P* < 0.05 was considered significant.

## Results

### General information and clinical outcomes

No complication was recorded such as venous thrombosis, pressure sores, and pneumonia. The skin incisions healed well, and the sutures were removed on time. All patients were followed up after the operation. The average follow-up period was approximately 16 ± 6 months for the K-rod internal fixation group, while it was approximately 17 ± 5 months for the PLIF group. During the follow-up period, no patients experienced screw loosening, pulling out, or broken nails. No significant difference was noted between the two groups in the preoperative data. Comparison of the perioperative data (such as the operation time, blood loss, postoperative drainage volume, postoperative exercise time turning to the ground, and postoperative hospital-stay duration) between the two groups revealed that the K-rod internal fixation group performed significantly better than the PLIF group (Table 1). The preoperative VAS score and the ODI for the K-rod group were 5.95 ± 1.18 and 59.87 ± 4.68, respectively; at the last follow-up, the corresponding values were 1.05 ± 0.62 and 9.37 ± 4.40, respectively. In the PLIF group, the preoperative VAS score and ODI were 6.53 ± 0.91 and 60.26 ± 7.45, respectively, while they were 2.53 ± 0.61 and 18.42 ± 3.75, respectively, at the final follow-up. An analysis of the difference in VAS and ODI between the K-rod and PLIF group showed that the main effects of the group, repetition, and interaction effect of repetition×group were all statistically significant (VAS: Group×Rep: *P* = 0.001, Group: *P* < 0.001, Rep: P < 0.001;ODI: Group×Rep: P < 0.001, Group: *P* = 0.003, Rep: P < 0.001). The detailed data were showed in Table 2. That is the comparison of the postoperative and preoperative VAS scores and ODI of the two groups at the last follow-up showed significant improvement over those before the operation and the VAS scores and ODI improvements were significantly better in the K-rod internal fixation group than in the PLIF group.

**Table 1:** Comparisons of basic characteristics of patients between K-rod group and PLIF

	K-rod group	PLIF group	*P* value
Gender (male/female)	21/6	13/12	0.051
Follow-up (month,^−^x ± s	16 ± 6	17 ± 5	0.000^***^
Operation time (min,^−^x ± s	144.7 ± 41.0	222.9 ± 86.1	0.000^***^
Blood loss (ml,^−^x ± s)	81.1 ± 48.5	294.0 ± 156.3	0.000^***^
Drainage (ml,^−^x ± s)	88.3 ± 44.1	212.9 ± 177.6	0.002^**^
Exercise time turning to the ground(d,^−^x ± s)	2.2 ± 0.5	5.2 ± 1.0	0.000^**^
Hospital stay time(d,^−^x ± s)	10.5 ± 2.7	15.0 ± 10.1	0.015^*^

*PLIF* Posterior lumbar interbody fusion. ^*^means *P* < 0.05.^**^means *P* < 0.01.^***^means *P* < 0.001

**Table 2:** Comparisons of VAS score and ODI of patients between K-rod group and PLIF (^−^x ± s)

	VAS		*P* value	ODI		*P* value
	Preoperative	postoperative		Preoperative	postoperative	
K-rod group	5.95 ± 1.18	1.05 ± 0.62		59.87 ± 4.68	9.37 ± 4.40	
PLIF group	6.53 ± 0.91	2.53 ± 0.61		60.26 ± 7.45	18.42 ± 3.75	
Group×Rep			0.001^**^			< 0.001^***^
Group			< 0.001^***^			0.003^**^
Rep			< 0.001^***^			< 0.001^***^

*PLIF* Posterior lumbar interbody fusion. *VAS* Visual analog scale. *ODI* Oswestry dysfunction index, ^**^means *P* < 0.01.^***^means *P* < 0.001

### Paraspinal muscle FCSA and paraspinal muscle fatty infiltration

At the final follow-up, the MRI revealed significant FCSA atrophy of the paraspinal muscles in the UAS and LAS of the two groups. However, the FCSA atrophies of the upper and lower adjacent paraspinal muscles in the K-rod group was significantly smaller than those in the PLIF group. The main effects of the group, repetition, and interaction effect of repetition×group were all statistically significant(*P* < 0.001). The detailed data were showed in Table 3. In addition, the patients who received the unilateral K-rod dynamic internal fixation showed no obvious paraspinal muscle atrophy on the surgical and contralateral side at the last follow-up in the paraspinal muscle FCSA of UAS and LAS. (UAS: Group×Rep: *P* = 0.001, Group: *P* = 0.446, Rep: *P* = 0.488;ODI: Group×Rep: *P* = 0.015, Group: *P* = 0.452, Rep: *P* = 0.278). The detailed data were showed in Table 4.

**Table 3:** Comparisons of paraspinal muscle FCSA of patients between K-rod group and PLIF (^−^x ± s)

	Upper segment FCSA (cm^2^)		*P* value	Lower segment FCSA		*P* value
	Preoperative	postoperative		Preoperative	postoperative	
K-rod group	10.74 ± 2.94	10.49 ± 2.93		15.13 ± 3.69	14.91 ± 3.47	
PLIF group	7.83 ± 2.55	6.97 ± 2.80		11.82 ± 2.69	9.82 ± 2.78	
Group×Rep			< 0.001^***^			< 0.001^***^
Group			< 0.001^***^			< 0.001^***^
Rep			< 0.001^***^			< 0.001^***^

*PLIF* Posterior lumbar interbody fusion. *FCSA* Functional cross-sectional area. ^***^means *P* < 0.001

**Table 4:** Comparison of UAS and LAS in the K-rod group between the surgical side and contralateral paraspinal muscle FCSA (^−^x ± s, cm^2^)

	UAS (cm^2^)		*P* value	LAS		*P* value
	Preoperative	postoperative		Preoperative	postoperative	
Surgical side	5.31 ± 1.46	4.98 ± 1.62		7.47 ± 2.04	7.11 ± 1.81	
Contralateral side	5.42 ± 1.51	5.64 ± 1.60		7.66 ± 1.72	7.81 ± 1.78	
Group×Rep			0.001^**^			0.015^*^
Group			0.446			0.452
Rep			0.488			0.278

*PLIF* Posterior lumbar interbody fusion. *FCSA* Functional cross-sectional area. ^*^means *P* < 0.05. ^**^means *P* < 0.01

An analysis of the difference in VAS between the K-rod and PLIF group showed that the area of fatty infiltration on UAS and LAS showed that the main effects of the group and repetition were all statistically significant (The area fatty infiltration on UAS: Group×Rep: *P* > 0.762, Group: *P* = 0.013, Rep: *P* = 0.001;The area fatty infiltration on LAS: Group×Rep: *P* = 0.429, Group: *P* < 0.001, Rep: *P* = 0.001). The detailed data were showed in Table 5. That is the fatty infiltration of the muscles in the UAS and LAS of the two groups increased. In addition, their fat cross-sectional area values were significantly increased when compared with those before the operation. The detailed data was showed in Table 5. Comparison of the distribution of paraspinal muscle fatty infiltration grade change value between the two groups at the final follow-up revealed that the K-rod internal fixation group showed significantly better outcomes than the PLIF group in the LAS (*P* = 0.002). The detailed data were showed in Table 6.

**Table 5:** Comparison the area of paraspinal muscle fatty infiltration between K-rod group and PLIF in UAS and LAS (^−^x ± s, cm^2^)

	Area of fatty infiltration on UAS		*P* value	Area of fatty infiltration on LAS		*P* value
	Preoperative	postoperative		Preoperative	postoperative	
K-rod group	1.91 ± 1.26	2.69 ± 1.46		3.17 ± 1.58	4.74 ± 2.27	
PLIF group	3.20 ± 1.92	4.13 ± 2.24		5.64 ± 1.84	8.12 ± 4.02	
Group×Rep			0.762			0.429
Group			0.013^*^			< 0.001^***^
Rep			0.001^**^			0.001^**^

*PLIF* Posterior lumbar interbody fusion. *UAS* Upper adjacent segment. *LAS* Lower adjacent segment

^*^means *P* < 0.05. ^**^means *P* < 0.01.^***^means *P* < 0.001.Rep:Repetition

**Table 6:** Comparison the changes in degree of paraspinal muscle fatty infiltration between K-rod group and PLIF in UAS and LAS

	K-rod group			PLIF group			*P* value
	0	1	2	0	1	2	
UAS	16	3	0	10	5	3	0.104
LAS	12	6	1	2	11	5	0.002^**^

*PLIF* Posterior lumbar interbody fusion. *UAS* Upper adjacent segment. *LAS* Lower adjacent segment. ^**^means *P* < 0.01

### Multivariate linear regression analysis of postoperative low back pain and paraspinal muscle atrophy and fatty infiltration

Bivariate correlation analysis showed a significant positive correlation was noted between the VAS score of postoperative low back pain and the area of fatty infiltration of UAS (r = 0.286, *P* = 0.043) and LAS (r = 0.506, *P* = 0.001). However, the negative correlation was observed between the VAS score of postoperative low back pain and the area of paraspinal muscle of UAS (r = − 0.590, *P* < 0.001) and LAS (r = − 0.563, *P* < 0.001). Multivariate linear regression analysis of the 4 risk factors showed that the main risk factors of low back pain contained the area of fatty infiltration of LAS (t = 2.738, *P* = 0.010) and the area paraspinal muscle of UAS (t = − 3.674, *P* = 0.01). But the area of fatty infiltration of UAS and the paraspinal muscle atrophy of LAS were not the main risk factors of low back pain. The detailed data were showed in Table 7. Regression equation: Y = 2.244–0.128X_1_ + 0.097X_2_ (X_1_ represents the area of paraspinal muscle of UAS; X_2_ represents the area of fatty infiltration of LAS).

**Table 7:** Correlational analysis of postoperative lower back pain and paraspinal muscle atrophy and fatty infiltration

Model	Unstandardized Coefficients		Standardized Coefficients	*t* value	*P* value
	B	Beta	Beta		
(Constant)	2.244	0.438		5.127	< 0.001^***^
Area paraspinal muscle of UAS(X_1_)	−0.128	0.035	−0.483	−3.674	0.001^**^
Area of fatty infiltration of LAS(X_2_)	0.097	0.035	0.360	2.738	0.010^*^

*UAS* Upper adjacent segment. *LAS* Lower adjacent segment. ^*^means *P* < 0.05.^**^means *P* < 0.01.^***^means *P* < 0.001

^a^ Dependent Variable: VAS score

## Discussion

Comparison of the clinical outcomes between the posterior K-rod dynamic internal fixation technique (based on the VAS score and ODI) with PLIF revealed that.

both the types of surgery can significantly improve the clinical symptoms. However, the clinical outcomes of the K-rod dynamic internal fixation were significantly better, owing to its shorter operation time, lesser blood loss, earlier exercise time turning to ground, and shorter hospital-stay duration. Furthermore, the posterior lumbar unilateral K-rod dynamic internal fixation showed significantly less paraspinal muscle atrophy and fatty infiltration than the PLIF group. Moreover, multivariate linear regression analysis suggested that postoperative low back pain was positively correlated with paraspinal muscle atrophy and fatty infiltration. These results confirmed our primary hypothesis.

The procedure of PLIF involves the disadvantages of a long surgical incision and relatively wider exposure that may lead to increased blood loss, postoperative.

drainage, and damage to the blood supply of the paraspinal muscles [25]. On the other hand, the K-rod dynamic internal fixation technique required only a small unilateral dissection, with a small incision. Moreover, the unilateral lamina of a single segment was required to be exposed on opening the lamina to remove the herniated intervertebral disc, which significantly shortened the operation time [23]. Furthermore, the paravertebral soft tissue and the blood supply were lesser damaged, such that the blood loss was lesser with lesser postoperative drainage. The posterior spinous process, interspinous ligament, and other structures of the spine could be preserved by this method, and the surgical method was effectively fixed dynamically. Thereby, the relevant functional activities could be performed earlier after the operation, which significantly shortened the postoperative hospital-stay duration. Recent studies have shown that both the methods can provide beneficial short-term effects [26, 27]. On the contrary, spinal fusion is performed for coping with lumbar instability in the PLIF procedure, which can be attributed to some complications such as adjacent segment diseases (ASD) [5, 6, 28], considering that the spinal fusion may change the rotation center over the disc, increasing the stress on the intervertebral disc of the adjacent mobile segments [29]. We also noted improvements in the VAS score and ODI at the last follow-up. In addition, the effect of K-rod dynamic internal fixation was significantly better than that of PLIF. The K-rod technology is performed to decrease the pressure on the intervertebral joint and to reduce its compensatory activity, which lowers the incidence of ASD [30]. This result may contribute to the improvement in the clinical outcome of the K-rod dynamic internal fixation technique. In this study, although the PLIF procedure could relieve the symptoms of low back pain and obtained satisfactory results, some patients continued to experience low back pain and muscle weakness after the operation.

The occurrence of lumbar paraspinal muscle atrophy and fatty infiltration is common after a posterior lumbar spine surgery, which can be mainly attributed to the direct injury of intraoperative dissection and traction that lead to paraspinal muscle ischemic necrosis and denervation. Some researchers have demonstrated that the posterior approach, open surgery, and interbody fusion are the main reason for the occurrence of paraspinal muscle atrophy and fatty infiltration after the surgery [31, 32]. Some past studies have reported that the use of retractor with a long stretch time may cause paraspinal muscle atrophy [8]. The damage to the posterior structure of the spine in the PLIF can inevitably cut off the attachment points of the paraspinal muscles on the spinous process and lamina. The scar formation of the postoperative muscle reduces the contraction function of the paraspinal muscles, resulting in postoperative paraspinal muscle atrophy [32]. Nevertheless, these drawbacks are rarely encountered with K-rod dynamic internal fixation [26]. The results obtained for the K-rod group showed no obvious muscle atrophy and fatty infiltration in the adjacent segments of the paraspinal muscles, which are consistent with the theoretical outcomes of a minimally invasive technique.

Presently, most scholars believe that the degree of postoperative residual low back pain is significantly related to paraspinal muscle atrophy and [30, 33, 34]. For instance, Crawford RJ et al. believe that paraspinal muscle atrophy is related to the degeneration of the intervertebral disc that results in chronic low back pain [33]. Shahidi B et al. also share the notion that low back pain and function related to paraspinal muscle atrophy and fatty infiltration [35]. Faur C et al. suggest that paraspinal muscle atrophy and fatty infiltration may be one of the key signs of low back pain [36]. In addition, MGT et al. believe that paraspinal muscle atrophy can be perceived as a predictor of the surgical outcome for spinal stenosis [37] . However, some other scholars contradict this viewpoint. For instance, He K report that postoperative residual low back pain is not always related to paraspinal muscle atrophy [38]. Especially, for lumbar disc herniation, no significant correlation was noted between postoperative lumbar back pain and paraspinal muscle atrophy with fatty infiltration. In this study, a significant positive correlation was confirmed between the residual low back pain and paraspinal muscle atrophy with fatty infiltration.

In summary, unilateral K-rod dynamic internal fixation under the channel is a safe and reliable minimally invasive technique to reduce postoperative paraspinal muscle atrophy and fatty infiltration, albeit its association with a slight damage to the paravertebral soft tissues. The proposed technology can significantly reduce the incidence of postoperative residual lower back pain and obtain satisfactory clinical outcomes.

However, several limitations were identified in the present study. First, the study was conducted as a retrospective analysis of a small sample size. Second, the follow-up period was short. The presence of these factors could have introduced some bias in the results obtained. A prospective clinical study or random control trail is warranted to estimate the clinical effect, paraspinal muscle atrophy, and fatty infiltration of posterior unilateral K-rod dynamic internal fixation.

## Data Availability

All data generated or analysed during this study are included in this published article.

## Abbreviations

PLIF: Posterior lumbar interbody fusion
LDD: Lumbar degenerative diseases
VAS: Visual analog scale
ODI: Oswestry dysfunction index
MRI: Magnetic resonance imaging
FCSA: Functional cross-sectional area
UAS: Upper adjacent segments
LAS: Upper and lower adjacent segments
ASD: Adjacent level spinal deterioration
LDH: Lumbar disc herniation
LSS: Lumbar spinal stenosis.
